# The effect of ultrasound treatment in combination with nisin on the inactivation of *Listeria innocua* and *Escherichia coli*

**DOI:** 10.1016/j.ultsonch.2021.105776

**Published:** 2021-10-07

**Authors:** Katherine M. Costello, Eirini Velliou, Jorge Gutierrez-Merino, Cindy Smet, Hani El Kadri, Jan F. Van Impe, Madeleine Bussemaker

**Affiliations:** a Department of Chemical and Process Engineering, University of Surrey, Guildford GU2 7XH, UK; b Centre for 3D Models of Health and Disease, Division of Surgery and Interventional Science, University College London, London W1W 7TY, UK; c School of Biosciences and Medicine, University of Surrey, Guildford GU2 7XH, UK; d BioTeC+ Chemical and Biochemical Process Technology and Control, KU Leuven Campus Gent, Gent, Belgium

**Keywords:** Ultrasound, Nisin, *L. innocua*, *E. coli*, Inactivation, Hurdle technology

## Abstract

Ultrasound, alone or in combination with natural antimicrobials, is a novel food processing technology of interest to replace traditional food decontamination methods, as it is milder than classical sterilisation (heat treatment) and maintains desirable sensory characteristics. However, ultrasound efficacy can be affected by food structure/composition, as well as the order in which combined treatments are applied. More specifically, treatments which target different cell components could result in enhanced inactivation if applied in the appropriate order. The microbial properties i.e. Gram positive/Gram negative can also impact the treatment efficacy.

This work presents a systematic study of the combined effect of ultrasound and nisin on the inactivation of the bacteria *Listeria innocua* (Gram positive) and *Escherichia coli* (Gram negative), at a range of cavitation conditions (44, 500, 1000 kHz). The order of treatment application was varied, and the impact of system structure was also investigated by varying the concentration of Xanthan gum used to create the food model systems (0 – 0.5% w/v). Microbial inactivation kinetics were monitored, and advanced microscopy and flow cytometry techniques were utilised to quantify the impact of treatment on a cellular level.

Ultrasound was shown to be effective against *E. coli* at 500 kHz only, with *L. innocua* demonstrating resistance to all frequencies studied. Enhanced inactivation of *E. coli* was observed for the combination of nisin and ultrasound at 500 kHz, but only when nisin was applied before ultrasound treatment. The system structure negatively impacted the inactivation efficacy. The combined effect of ultrasound and nisin on *E. coli* was attributed to short-lived destabilisation of the outer membrane as a result of sonication, allowing nisin to penetrate the cytoplasmic membrane and facilitate cell inactivation.

## Highlights

• A multi-frequency study of ultrasound (US) and nisin for microbial inactivation.
• US impacts *E. coli* at 500 kHz only; *L. innocua* resists all frequencies studied.
• Nisin applied before US enhances inactivation of *E. coli* but not when applied after.
• Attributed to outer membrane destabilisation by US allowing nisin penetration.
• System structure (viscosity) reduces US inactivation efficacy.

## 1 Introduction

Ultrasound is currently used in the food industry for a variety of applications, such as emulsification, mixing, homogenising, and enzyme activation [1], [2]. Ultrasound for microbial inactivation has the potential for application to minimally-processed foods [3], [4] since ultrasound is generally milder than traditional heat inactivation methods, and these foods can therefore retain sensory and nutritional characteristics [3], [4], [5]. The mechanism for microbial inactivation by ultrasound is generally accepted to be as a consequence of ultrasound transient cavitation (for example, [6], [7], [8], [9]). More specifically, microorganism inactivation can be attributed to cell membrane damage from extreme temperature hot-spots or high pressures from bubble collapse, which can also further enhance damage by reactive species [10], [11]. High shear forces on the cell membrane as a result of microstreaming can also cause damage [12], [13], [14]. Furthermore, chemical effects such as the formation of free radicals (examples include ^•^OH, O^•^, H^•^, H_2_O_2_, and HO_2_) can cause external and internal cell damage [6]. These components are already known individually to have a bactericidal effect in systems unrelated to ultrasound [15], [16].

Ultrasound has potential as a hurdle technology (i.e. a technology that ensures elimination or control of food pathogens by providing multiple hurdles to survival) when combined with other non-thermal technologies, including natural antimicrobials such as nisin [7], [17], [18]. Nisin is generally recognised as safe by the Food and Drug Administration (FDA), and is one of the few natural antimicrobials approved by the European Union for use in foods: it is currently used as a preservative to prevent the growth of microorganisms, particularly in dairy products and acidic foods [19], [20], [21], [22]. The main inactivation pathway by nisin results from pore formation in the cell wall following the binding of nisin to Lipid II in Gram positive bacteria. Nisin is not effective against Gram negative bacteria, due to the different structure of the cell wall [11], [21]. We propose that ultrasound combined with nisin treatments may result in activity of nisin against Gram negative species, as ultrasound could disrupt the cell wall and allow for nisin penetration. This treatment combination may lead to synergistic activity.

The order in which treatments are applied could impact their efficacy and the microbial response, depending on the target in the cell i.e. a treatment which damages the outer membrane followed by one which acts on internal components of the cell could result in enhanced inactivation [11], [23]. However, both ultrasound and nisin treatments, alone or combined, like other minimal processing approaches, are milder than classical decontamination methods and may therefore present a mild, sublethal stress allowing for post-treatment survival and potential antimicrobial resistance (AMR) development [5], [24], [25], [26], [27], [28]. This is especially concerning for pathogenic bacteria commonly associated with ready-to-eat food products such as *Listeria* spp. and *E. coli*. Furthermore, the mechanisms of resistance to nisin through modification of the cell wall can lend resistance to antibiotics with a similar mode of action, meaning that typical treatments for listeriosis (for example) may not be as effective [29], [30]. In particular, reports of AMR in *Listeria* species isolated from the environment have become more prevalent in recent years, which is of huge concern due to the high mortality rates associated with listeriosis [31], [32], [33], [34], [35].

The chemical composition and rheological/structural properties of liquid and solid(like) foods can vary greatly, even between liquid food products. This can impact the efficacy of ultrasound treatment: bubbles become more stable as the system viscosity increases and are less likely to collapse, potentially affecting the transient cavitational activity [36], [37], [38]. As noted above, transient cavitation is believed to be one of the main mechanisms for microbial inactivation, thus the efficacy of inactivation treatment could be impacted. The effect of viscosity on bubble oscillation and cavitation has been investigated both theoretically and experimentally [36], [37], [38], [39], [40], [41], [42], [43]. However, a systematic study on the effect of viscosity on ultrasound inactivation of bacteria has not yet been conducted.

The efficacy of ultrasonic cavitation can be impacted by many other factors including frequency, applied power, sonication time, system volume, pressure amplitude, type of transducer i.e. equipment set-up, probe or plate diameter, liquid height and liquid temperature [44], [45]. Most studies on microbial inactivation are conducted at low frequencies (20–44 kHz) and high powers (>100 W applied power), with few studies conducted at higher frequencies (>100 kHz) or low power [8], [13], [46], [47]. Sonochemical effects in general are enhanced in the range 200–1,000 kHz thus the inactivation of microorganisms could also be enhanced at higher frequencies [45], [48], [49]. The effect of Gram-stain on ultrasound efficacy is yet to be fully elucidated, with some studies indicating that Gram positive bacteria (e.g. *Listeria*) are more resistant to ultrasound than Gram negative bacteria (e.g. *E. coli*) [12], [50], [51], [52], [53], while others identify no difference in efficacy between Gram positive and Gram negative species [8], [13], [14], [54].

Ultrasound inactivation of microorganisms both alone and in combination with antimicrobials (e.g. essential oils, chlorine dioxide, fumaric acid) has been studied in a range of liquid systems such as wastewater, saline solution, and growth medium [4], [14], [47], [55], [56], in real liquid food systems i.e. fruit juices, cactus pear juice, liquid whole egg or milk [17], [18], [57], [58], [59], [60], on the surface of solid ready-to-eat food products i.e. lettuce leaves, strawberries, cherry tomatoes, bean sprouts [61], [62], [63], [64], and also on abiotic surfaces such as stainless steel [65], [66]. However, the frequency, power, and experimental set-up vary significantly between studies, with very few groups investigating microbial inactivation at higher frequencies [8], [13], [46]. The system viscosity also varies significantly between these studies, which could impact the treatment efficacy. Furthermore, to the authors’ best knowledge, very few studies investigate the combination of ultrasound and nisin for microbial inactivation, with the available studies conducted for different experimental set-ups, systems, frequencies, powers and species [11], [17], [67], [68]. As such, a fundamental systematic study of the effect of system viscosity on microbial inactivation by ultrasound, at a range of frequencies and in combination with natural antimicrobials i.e., nisin, is lacking.

This study aims to identify the effect of ultrasound at a range of frequencies, alone and in combination with a sublethal concentration of nisin, on *Listeria innocua* (Gram positive) or *Escherichia coli* (Gram negative), in systems of varied viscosity.

## 2 Materials and methods

### 2.1 Preparation of viscoelastic model systems

Xanthan gum (XG) was selected as the gelling agent in this study as it is stable at a wide range of temperatures and is widely used in the food industry as a thickener and stabiliser in food products such as dressings, sauces, and bakery items [69], [70]. Viscoelastic XG systems were developed using a modified method of Velliou et al. [71]. Briefly, XG (Xantural® 75; CP Kelco, UK) was added to Tryptic Soy Broth supplemented with 0.6% Yeast Extract (TSBYE) at concentrations of 0.1%, 0.3%, or 0.5% w/v and mechanically stirred for at least 5 min until fully homogenised (Omni Mixer Homogenizer, Omni International Inc., USA). The homogenised mixture was centrifuged at 4000 × *g* in 50 mL falcon tubes (Corning Inc®, USA) for at least 30 min for removal of entrapped air bubbles (Megafuge 16R, ThermoFisher, USA). After autoclaving at 121 °C for 15 min, the viscoelastic medium was centrifuged again to remove additional air bubbles.

### 2.2 Rheological characterisation of the viscoelastic models

The rheological stability of the XG systems was tested at 37 °C, the optimum growth temperature for both species, by conducting dynamic oscillatory measurements to assess the viscoelastic behaviour. More specifically, the frequency dependence and magnitude of the storage modulus *G’* and loss modulus *G”* were measured. *G’* represents the elastic portion of the material response (somewhat a measure of the stiffness of a gel), while *G”* represents the viscous response. The loss tangent (*tanδ* = *G”*/*G’*) indicates whether the material is closer to an elastic solid (*tanδ* < 1), or a viscous fluid (*tanδ* > 1).

*G’* and *G”* values were obtained over a frequency range of 1–100 rad/s using a rotational Physica MCR 200 rheometer (Physica MCR 200, Anton Paar GmbH, Germany), with the temperature regulated using a Paar Physica circulating water bath (Viscotherm VT2, Anton Paar GmbH, Germany). A cone and plate geometry was used (50 mm diameter, 2° angle) and at least two independent replicates of at least two samples were analysed for each viscoelastic system. The strain was kept constant at 2%.

### 2.3 Inoculum preparation

Stock cultures of *L. innocua* ATCC 33090, a model for the foodborne pathogen *L. monocytogenes*, and of *E. coli* ATCC 25,922 were stored at −80 °C in TSBYE (Oxoid Ltd., UK), supplemented with 15% glycerol. A loopful of thawed culture was inoculated in 15 mL TSBYE for 9.5 h at 37 °C, the optimum temperature for growth. 20 µL was subsequently transferred to fresh 15 mL TSBYE and cultured for 15 h at 37 °C until early stationary phase was reached (10^9^ CFU/mL). Each species was cultured to stationary phase separately.

### 2.4 Ultrasound experimental set-up

Interchangeable piezoelectric transducers of 44 kHz, 500 kHz and 1000 kHz (Honda Electronics), comprising a 5 cm diameter piezoelectric ceramic round adhered to a 10 cm diameter vibration plate, were screwed onto the base of a custom-made plastic box (12 × 12 × 16 cm). The contact area between the transducer plate and the liquid carrier was 7 cm diameter. An amplifier (AG 1006 amplifier, T&C Power Conversion Inc., USA) was connected via (i) an impedance matching device (IMD) and (ii) a discharge plug to the transducer. The IMD and discharge plug were used to minimise the reflected power (RP) to the amplifier, alongside frequency tuning as required (Table 1). The applied power was 30 W for all frequencies. These frequencies were selected to represent a range of cavitation conditions while keeping experimental parameters constant, such as sample vial position. As such, the experimental set-up remained constant for all frequencies.

**Table 1:** Calorimetric powers for frequencies 44, 500 and 1000 kHz at 30 W. Each frequency was tuned to minimise the RP value, which occurs as a result of poor impedance matching. The standard deviation of measured values is indicated.

**Frequency (kHz)**	**Applied Frequency (kHz)**	**RP (W)**	**Calorimetric Power (W)**	**Calorimetric Power (W/mL)**	**Maximum Temperature (**°**C)**
44	44.7	4	17.8 ± 0.8	3.49 × 10^-2^ ± 0.002	37.7 ± 1.7
500	512.0	0	19.1 ± 1.6	3.74 × 10^-2^ ± 0.002	43.6 ± 0.5
1000	1000.6	0	20.3 ± 0.9	3.99 × 10^-2^ ± 0.003	45.9 ± 1.0

Borosilicate scintillation vials (Fisher Scientific, UK) were used to hold the biological samples, which contained a constant volume of 10 mL. Any variation between vial position/wall thickness is accounted for in the measured experimental error. The sample bottle was suspended in 500 mL of degassed deionised water inside the reactor vessel, aligned vertically over the centre of the transducer plate at a height of 1.7 cm above the plate, with the liquid level inside the vial aligned with the liquid level in the vessel (see Fig. S1 for an experimental schematic and further dimensions). This location was selected following dosimetry tests to identify the optimum sample elevation above the transducer at 500 kHz (results not shown). As this height was to be maintained at all frequencies for consistency, this test was not repeated at 44 kHz or 1000 kHz. Degassed water was used to prevent bubbles being trapped beneath the vial and was replaced every 30 min with fresh degassed deionised water. Trapped bubbles were also watched for during treatment and the experiment discarded if any appeared.

This experimental set-up was selected to minimise the risk of contamination of the microbial samples, as per previous studies [8], [51], [53]. This was crucial to ensure the response of the single species was observed, as inter-species interactions and communication are known to cause different microbial responses and growth patterns to when species are cultured alone [72], [73].

The temperature effect of sonication on microbial inactivation was tested by heating samples in a water bath for 30 min (with and without nisin) to the maximum temperature obtained of 46 °C (Table 1), thus simulating the temperature increase following sonication (results not shown). No significant reduction (CFU/mL) was observed for the treated samples thus any observed inactivation was not due to ultrasound-induced temperature increase.

### 2.5 System quantification using calorimetry and KI dosimetry

For calorimetric quantification, 10 mL distilled water was used in the place of a biological sample with a temperature probe suspended in the centre of the vial. The system was sonicated at 44 kHz, 500 kHz or 1000 kHz with an applied power of 30 W and the temperature recorded every 30 s for 15 min of sonication. The calorimetric power (Table 1) was calculated as per the method of Koda et al. [74] using the equation:

$$Power\left(W\right)=\left(\frac{\mathrm{dT}}{\mathrm{dt}}\right)c_{p}M$$

where *c_p_* is the specific heat capacity of water (4.2 J/Kg), *M* is the total mass of water (g), and *dT/dt* is the temperature rise per second [74]. The density of water was taken as 1 g/cm^3^ and the total mass of water was 510 mL i.e. the reactor vessel contents (500 mL) plus the sample vial volume (10 mL).

KI dosimetry was conducted as per the method of Koda et al. [74]. Briefly, this method works on the principle that when an aqueous potassium iodide (KI) solution is sonicated, I^-^ ions are oxidised to give I_2_. Excess I^-^ ions present in the solution can react reversibly with I_2_ to produce the I_3_^-^ ion, which appears yellow in solution: the change in colour can be used to indicate the concentration of I_3_^-^. A 0.1 mol/L KI solution was prepared with deionised water. 10 mL KI solution was sonicated at 44 kHz, 500 kHz or 1000 kHz with an applied power of 30 W for up to 15 min. The absorbance of I_3_^-^ at 355 nm was recorded and the I_3_^-^ concentration calculated using the Beer-Lambert absorption law (path length = 1 cm, molar absorptivity = 26303 L/mol/cm) [74]. For both quantification methods, at least three independent repeats were conducted with fresh samples and fresh degassed water in the reactor vessel.

### 2.6 Ultrasound inactivation

**Fig. 1:**
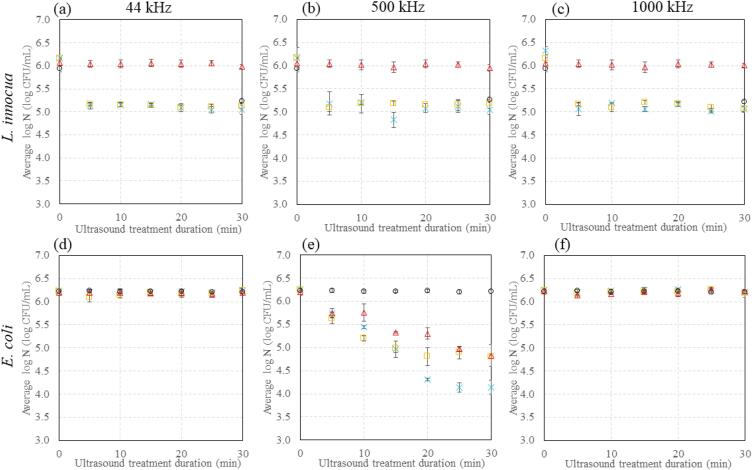
Inactivation kinetics of (a-c) *L. innocua* and (d-f) *E. coli* following nisin and/or ultrasound treatment at (a,d) 44 kHz, (b,e) 500 kHz, or (c,f) 1000 kHz, in liquid nutrient broth. In all plots, (○) nisin only, () ultrasound only, () 30 min nisin followed by 0 – 30 min ultrasound (5 min increments), () 0 – 30 min ultrasound (5 min increments) followed by 30 min nisin. Each point is the average of three independent experiments with two replicate samples per experiment. Error bars indicate the standard deviation. Sample vial height was maintained at 1.7 cm above the transducer plate for all frequencies.

*L. innocua* or *E. coli* was cultured and grown to stationary phase in 15 mL TSBYE at 37 °C, as previously described [25], [75], [76]. The culture was serially diluted in TSBYE then inoculated in (i) TSBYE, (ii) 0.1% XG, (iii) 0.3% XG or (iv) 0.5% XG to a final cell density of 10^6^ CFU/mL. 10 mL of these systems was transferred into a borosilicate scintillation vial. Samples remained at room temperature (approx. 20 °C) for a maximum of 1 h before ultrasonic treatment for 0 – 30 min at either 44 kHz, 500 kHz or 1000 kHz, and were removed immediately to an ice bath for a maximum of 2 h before sample processing. Samples were serially diluted in TSBYE and plated on tryptic soy agar supplemented with 0.6% YE (TSAYE) plates for enumeration of both species. Plates were incubated at 37 °C for 24 h before enumeration (CFU/mL). To maintain a constant sample volume during ultrasound treatment of up to 30 min, samples were sonicated for the required duration (0, 5, 10, 15, 20, 25, 30 min) then sacrificed for processing. Each point presented in Fig. 1 and Fig. 7 is the average of three independent experiments with two replicate samples per experiment.

### 2.7 Combined inactivation: Ultrasound and nisin

10 mL samples of *L. innocua* or *E. coli* at a cell density of 10^6^ CFU/mL were prepared in 0%, 0.1%, 0.3% and 0.5% XG systems, as previously described for ultrasound inactivation. The nisin treatment time was fixed at 30 min, and was applied either before or after ultrasound treatment, which was varied between 0 and 30 min. Three independent experiments with two replicate samples per experiment were conducted.

Nisin was applied at a concentration of 35 IU/mL, which was selected as it resulted in a 1 log reduction of *L. innocua* (in order to observe the combined effect with ultrasound, data not shown). This test was not performed on *E. coli* as nisin is not effective on Gram negative species [21].

### 2.8 Sonochemiluminescence

Solutions of 0%, 0.1%, 0.3% and 0.5% XG containing 1 mM luminol (5-Amino-2,3-dihydrophthalazine-1,4-dione) (Sigma Aldrich) and 0.1 M NaOH (Sigma Aldrich) were prepared for use immediately prior to sonochemiluminescence (SCL) and kept protected from light until use. The sonication equipment was moved into a dark room in order to detect and capture the released luminescence, which is not visible by the naked eye. 10 mL samples were sonicated as previously described for inactivation experiments. An ANDOR iXon3 EMCCD camera, operating at – 70 °C, and software was used to capture images of the systems after 30 s of sonication, with an applied EM gain level of 15 and exposure time of 1 s. Raw images were exported as 8-bit TIFF-files and the intensity value calculated using ImageJ 2.0 [77].

### 2.9 Flow cytometry

Flow cytometry analysis was conducted to obtain an indication of the level of injury to the overall population rather than identifying only the dead cells (i.e. inactivation kinetics based on colony forming unit (CFU) counts on TSAYE), to gain a better understanding of ultrasound mechanisms, and to further characterise the effects of sonication. Propidium iodide (PI) and bis-(1,3-dibutylbarbituric acid) trimethaine oxanol (BOX) (Sigma-Aldrich, UK) fluorescent stains were used to visualise the dead population and the injured population respectively. PI enters cells that have lost their membrane integrity (i.e. dead cells) and binds to nucleic acids, giving a bright red fluorescence. BOX enters depolarised cells, i.e. where the membrane potential is reduced to zero, binding to the membrane or intracellular proteins to give a bright green fluorescence. Cell depolarisation is an indication of cell injury [78].

Flow cytometry analysis was conducted using an Attune NxT flow cytometer (Thermo Fisher Scientific, UK). A 0.5 mL sample was stained with BOX (100 ng/mL) and PI (4 µL/mL) and incubated at room temperature for 5 min. The stained samples were excited using a 488 nm solid state laser and fluorescence was detected using 530/30 BP (BL1 channel) and 620/15 BP (YL2 channel) filters corresponding to BOX and PI fluorescence respectively. Approximately 10,000 data points were collected and the data was analysed using Attune NxT software. Three independent experiments were performed with two replicates each time.

### 2.10 Scanning Electron microscopy

Scanning Electron Microscopy (SEM) was conducted on liquid samples following ultrasound treatment to identify any impact of ultrasound at a cellular level. 10 mL samples were sonicated for 30 min and the cells were collected by centrifugation at 4500 × *g* for 10 min at 4 °C. The cell pellet was resuspended in 1 mL sterile Dulbecco’s Phosphate Buffered Saline (DPBS), modified without CaCl and MgCl_2_, then 1 mL of 3% v/v formaldehyde solution was added to the resuspended cells and incubated for 1 h at room temperature to fix the cells. 3 mL DPBS was added to the total fixed sample to make a total volume of 5 mL.

A sterile 13 mm diameter polycarbonate track-etched membrane filter (0.22 µm, GE Healthcare Whatman, Fisher Scientific, UK) held in a sterile Swinnex filter holder (13 mm, Merck, USA) was wetted with 2 mL DPBS then the fixed cell suspension was passed slowly through the filter. The filter was removed to a sterile petri dish and dehydrated in an ethanol in MilliQ series (20, 40, 60, 80, 100% v/v ethanol) for 10 min at each stage, repeating the 100% stage twice. The samples were air dried overnight and subsequently transferred to a desiccator for 12 h. Filters were mounted onto aluminium stubs using silver paint at each corner and gold sputter coated in a vacuum evaporator (EMITECH K575X, Quorum Technologies, Lewes). Samples were visualised using a JSM-7100F Field Emission SEM (JEOL Ltd).

### 2.11 Statistical analysis

To ensure statistical significance, at least three independent experiments were conducted with two replicate samples throughout. Quantitative results were collected in Microsoft Excel and the mean, standard deviation and standard error were calculated for each replicate result. Student’s *t*-test was used to compare two mean values. Differences were considered significant at P < 0.05. Standard deviation is reported as error bars on the plots.

## 3 Results and discussion

### 3.1 The inactivation efficacy of individual treatments for *L. innocua* and *E. coli*

Fig. 1 displays the inactivation kinetics of *L. innocua* and *E. coli* treated with ultrasound at 44 kHz, 500 kHz, and 1000 kHz, in combination with or without artificial nisin (35 IU/mL) added for 30 min before or after ultrasound treatment, in liquid TSBYE. The combination of ultrasound and nisin will be discussed in Section 3.2.

#### 3.1.1 Inactivation of *L. innocua* by individual treatments

For *L. innocua*, no effect of ultrasound is observed at any frequency, although an effect of the added nisin is observed at all frequencies (Fig. 1a-c). More specifically, as can be seen in Fig. 1a-c, treatment with ultrasound alone (red symbols) did not result in a reduction in cell count at all three frequencies studied. For treatment with nisin alone (black symbols), a 1-log reduction is observed at all frequencies (Fig. 1a-c). The effect of nisin is expected, as it is widely known to act on *Listeria* species [25], [75], [76], [79], [80]. The lack of effect of ultrasound suggests that *L. innocua* is resistant to ultrasound treatment under the conditions studied. Generally, Gram positive bacteria are suggested to be more resistant to ultrasound than Gram negative bacteria due to their thicker cell wall [12], [50], [51], [52], [53], [81]. Furthermore, *Listeria* species are known to have an innate resistance to nisin through mechanisms such as cell wall thickening [20], [30], [82] which could also lend resistance to other inactivation technologies which may also act on the cell wall, such as ultrasound. Indeed, some previous studies also observe resistance of *Listeria* species to ultrasound. More specifically, ultrasound alone had no effect when the combination of (i) nisin, (ii) ultrasound (20 kHz, 60 W applied power, horn configuration, 2.5 min) and (iii) high-pressure treatments on *Listeria seeligeri* in liquid whole egg was studied [67]. Similarly no effect of ultrasound was observed at 20 kHz, 750 W applied power, horn configuration, on *L. monocytogenes* 10403S inactivated in saline solution for 20 min [83]. However, other studies did observe an impact of ultrasound on *Listeria*: for example Muñoz et al. [68] report a 1-log reduction for *L. innocua* treated with ultrasound in a transparent buffer system (no nutrients) (20 kHz, applied power 500 W, continuous flow with residence time = 126 s), and Inguglia et al. [51] show a 2-log reduction for *L. innocua* treated with ultrasound in TSB (20 kHz, 75 W applied power, horn configuration, up to 60 min) [51], [68]. Further studies observe similar inactivation [4], [47], [62], [84]. It is noted that the majority of these studies are conducted at 20 kHz, and their experimental set-up varies to the present study, however they are used for comparison with the present study as to the authors’ best knowledge no studies exist at the present frequencies for the inactivation of *Listeria* species. Furthermore, the differences in efficacy reported in the literature are likely due to the variations in power, experimental set-up, treatment time and intensity, and the strain of bacteria used: variations in resistance to ultrasound between strains of *Listeria* has been reported [81], [83]. As such it cannot be concluded that the ultrasound settings used in the present study are inappropriate for the inactivation of all *Listeria* species, only that the strain *L. innocua* ATCC 33,090 (used in this study) is resistant to these settings.

#### 3.1.2 Inactivation of E. coli by individual treatments

Inactivation of *E. coli* by ultrasound alone is observed at 500 kHz only, i.e. a 1-log reduction in cell count is observed at this cavitation condition and not at the other conditions studied (Fig. 1 d–f, red symbols).

**Fig. 2:**
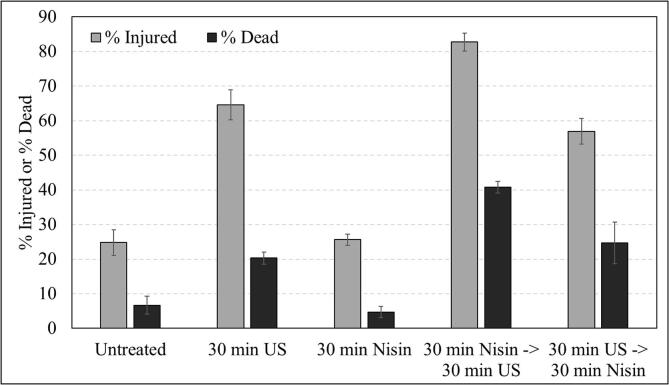
Flow cytometry analysis of treated and untreated *E. coli* cells with 500 kHz ultrasound (30 min), and/or 35 IU/mL nisin (30 min). The injured and dead populations are identified by BOX and propidium iodide fluorescent stains respectively. Error bars indicate the standard deviation.

Fig. 2 presents flow cytometry data for *E. coli* treated with ultrasound at 500 kHz and/or nisin, which identifies the injured and dead population in each treated system. An increase in the % injured and % dead populations is also observed in the flow cytometry analysis following ultrasound treatment (Fig. 2).

*E. coli* is previously shown to be more susceptible to ultrasound treatment than Gram positive species [8], [52], [53], [67], [81], [85]. As discussed previously, this difference is likely due to the Gram stain of the species. Gram negative cells such as *E. coli* have a thinner cell wall than Gram positive cells, providing less protection against ultrasonic effects and thus explaining the differences in efficacy observed for *L. innocua* and *E. coli* in the present work [12], [50], [51], [52], [53], [81].

#### 3.1.3 Frequency effects on E. coli inactivation by ultrasound only

As noted in the previous section, inactivation of *E. coli* (but not *L. innocua*) was observed at 500 kHz only and not at 44 kHz or 1000 kHz (Fig. 1d–f). Furthermore, from system characterisation with KI dosimetry, the concentration of I_3_^-^ was much greater at 500 kHz than at the two other frequencies studied, indicating a greater sonochemical activity at this cavitation condition (Fig. S2, Supplementary Information). A greater intensity was also observed through SCL imaging at 500 kHz as compared to 44 kHz and 1000 kHz (Fig. S3, Supplementary Information). It is noted that similar SCL, KI dosimetry, and calorimetry trends were observed in our previous work where direct sonication was applied without bacteria [86]. As such, given the challenges of performing direct sonication (discussed in the Materials and Methods), the indirect method used in the present study is a suitable proxy for direct sonication.

Sonochemical effects in general are enhanced in the range 200–600 kHz [45], [48], [49] which could explain why ultrasound treatment is most effective for *E. coli* at 500 kHz as compared to 44 kHz and 1000 kHz. *E. coli* inactivation in saline solution was slightly enhanced at 500 kHz as compared to at 20 kHz (plate and horn configuration, respectively, applied power 1.7–12.4 W) with inactivation at 500 kHz attributed to a combination of physical and chemical effects of ultrasound [8]. The lack of inactivation observed in our work at 1000 kHz is likely due to an increased cavitational threshold and reduced physical effects of ultrasound [45], [87]. Indeed, erosion of aluminium foil by physical effects of acoustic cavitation (plate, applied power starting at 1 W) was observed at frequencies between 22 kHz and 488 kHz, but not at 1000 kHz, due to the higher cavitation threshold as frequency increases [88]. It is therefore suggested that the mechanism of ultrasound action at this cavitation condition requires a physical aspect, congruent with the disruption of the cell membrane with ultrasound that can also augment nisin treatment. Chemical effects may also play a part, as evidenced by the SCL and KI activity (Figs. S2 and S3).

The lack of inactivation at 44 kHz in the present work is attributed to the relatively low power compared to previous studies. Another group using a similar plate experimental set-up also observed no microbial reduction for the treatment of abattoir wastewater at 44 kHz and applied power 40 W [89]. However, inactivation of *E. coli* was observed following ultrasonic treatment at 40 kHz and 600 W, using a bath configuration [61]. Inactivation of *E. coli* and *S. aureus* was also observed at 30 kHz and 100 W with a horn configuration [90]. Other studies conducted at low frequencies (20–44 kHz) and high powers also report microbial inactivation for *E. coli* [51], [55], [63], [91].

It is also noted that erosion of aluminium foil was observed at 43 kHz and 1 W in a plate system, although for an irradiation time of 180 min [88]. This suggests that, while physical effects may exist at 44 kHz, a much longer sonication time is required for the effects to be observed in the present system. As discussed in the Materials and Methods, the maximum sonication time was 30 min, after which the carrier liquid (water) required further degassing. It is therefore suggested that fewer cavitation events and a weaker bubble collapse is experienced at 44 kHz in comparison to previous literature, resulting in reduced physical effects and the formation of fewer radicals for chemical effects.

As noted in the Materials and Methods, the experimental set-up was kept constant at each frequency condition. As such, it is possible that activity at 44 kHz and 1000 kHz might be observed if the set-up is optimised for each frequency applied, however this was out of the scope of this work and will be addressed in our future work (see the Conclusions).

### 3.2 The inactivation of *L. innocua* and *E. coli* by nisin and ultrasound combined treatments

#### 3.2.1 Inactivation of *L. innocua*

No effect of the combination of ultrasound and nisin is observed on *L. innocua* at any frequency, regardless of the order of application, as also observed for individual treatments (Fig. 1a-c, blue and yellow symbols). More specifically, the cell count is not reduced in any system for the combined treatments (other than the expected effect of nisin). As previously discussed in Section 3.1.1., this is attributed to an innate resistance of the *Listeria* strain used in this study.

#### 3.2.2 Inactivation of *E. coli*

Differences in inactivation efficacy of *E. coli* are observed for the different treatment combinations at 500 kHz, with no effect of combined treatments at the other cavitation conditions (as noted for individual treatments in Section 3.1.2.) (Fig. 1d-f). More specifically, a 1-log reduction is observed for the combination of nisin added after ultrasound treatment, which is no different to the reduction following ultrasound treatment alone (Fig. 1e). In addition, the injured and dead populations are not statistically different for these conditions, with no significant visual differences on a cellular level as compared to ultrasound treatment alone (Fig. 2). However, a 2-log reduction is achieved for the combination of nisin added before ultrasound treatment (Fig. 1e), and a greater level of injury and death is observed in the flow cytometry data (Fig. 2).

#### 3.2.3 Microscopic effects of ultrasound treatment, alone or with nisin, on *E. coli*

**Fig. 3:**
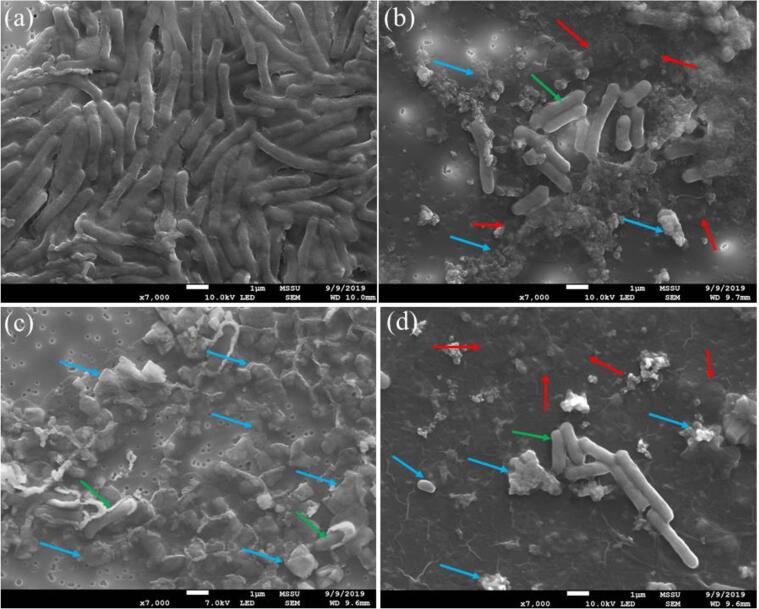
Scanning Electron Microscopy images of *E. coli* cells: (a) untreated, (b) after 30 min ultrasound at 500 kHz, (c) after 30 min nisin then 30 min ultrasound at 500 kHz, and (d) after 30 min ultrasound at 500 kHz followed by 30 min nisin. Examples of intact cells (green arrows), ghost cells (red arrows), and cellular debris (blue arrows) are indicated. (For interpretation of the references to colour in this figure legend, the reader is referred to the web version of this article.)

Fig. 3 presents scanning electron microscopy (SEM) images of *E. coli* cells, treated with and without ultrasound (500 kHz) and nisin. The effect of ultrasound treatment on a cellular level at this frequency is clear from the SEM images: far fewer intact cells are observed for ultrasound treatment (Fig. 3b) in comparison to the untreated control (Fig. 3a). “Ghost cells” are observed alongside cellular debris (Fig. 3b, red arrows). In these images the ghost cells are flattened, most likely due to the force necessary to collect the cells by filtration before imaging.

Ghost cells are empty (dead) cell envelopes lacking in cytoplasmic content but retaining their morphological and structural features [92]. Expulsion of the cytoplasmic content occurs due to high osmotic pressure [93]. It is therefore likely that the mechanism for the formation of ghost cells is related to mechanical effects of ultrasound and the localised pressure shockwave created on bubble collapse and/or sonoporation effects from microstreaming. Indeed, the pressure shockwave has been suggested to be more important for the inactivation of *E. coli* compared to chemical effects [94].

Cellular debris consists of cellular contents that have been released or expelled through the membrane following cell death: the presence and quantity of cellular debris therefore provides an indication of the occurrence and level of cell death. As such, the presence of both ghost cells and cellular debris following ultrasound treatment (Fig. 3b) suggests that the mechanism of inactivation by ultrasound in this system involves the destruction or disturbance of the cell membrane, causing efflux of the cell contents and the death of the cell – but not full destruction of the cell membrane, as evidenced by the presence of ghost cells.

Empty cell envelopes of *E. coli* following sonication at 500 kHz (applied power 12.4 W), were also observed by Koda et al. [8] and the mechanism of inactivation attributed to damage to the cell membrane following radical attack [8]. Further studies also report the presence of ghost cells following ultrasound on a range of biological samples including *E. coli*, *L. innocua*, *S. aureus,* blood cells, and yeast [6], [46], [90], [95], [96], [97]. Many other studies also show evidence of cell breakage and debris following ultrasound treatment at a range of frequencies, further lending to the theory that inactivation by ultrasound involves the disruption of the cell membrane through various mechanisms such as pore formation, mechanical disruption by pressure shock waves, jetting and shear effects [6], [46], [55], [98], [99], [100], [101].

It is noted that nisin alone has no effect on *E. coli* (Fig. 1d-f, Fig. 2), as expected: nisin is known to be ineffective against Gram negative species due to the presence of the outer membrane, which prevents nisin from reaching Lipid II in the cell membrane [21]. As such, SEM imaging was not conducted for this condition in the present study.

For the combination of nisin added before ultrasound treatment, a greater quantity of cellular debris is observed for the combination condition compared to the other treatments (Fig. 3c), with fewer intact cells present, and no ghost cells. The lack of ghost cells and significant quantity of cellular debris suggests that this combination of treatments causes complete cell rupture and disintegration of the cell wall.

Nisin forms pores in the cell membrane [30], [102], [103]which, combined with localised high pressure and shock waves from cavitation, could cause the cell to disintegrate into many small sections, thus explaining the present observations (Fig. 3c). Very few studies have investigated the combination of nisin with ultrasound [11], [17], [67], [68]: none of these studies identify on a microscopic level the combined effects of both treatments. This is the most effective treatment in this system, despite the fact that nisin alone does not affect *E. coli* cells (Fig. 1d-f, Fig. 2).

As such we propose that, in addition to the terminal damage to the cell wall and membrane, ultrasound may also cause short-lived damage to the outer membrane of *E. coli* cells, which is reversed or fixed when ultrasound is ceased. We suggest that this allows the nisin present in the system access to the cell membrane where it can bind to Lipid II, which it would otherwise not be able to interact with (due to the presence of the usually intact outer membrane). Under certain processing conditions, ultrasound can destabilise the outer membrane of *E. coli* without rupturing the cytoplasmic membrane [52], [104]. This destabilisation would allow the penetration of nisin to the cytoplasmic membrane and thus facilitate cell inactivation through this mechanism.

Elsewhere the inactivation of *E. coli* by ultrasound (20 kHz, 190 W applied power, horn configuration) and nisin, added immediately before ultrasound treatment (thus it was present during sonication, as in this study) saw a ∼0.5 log reduction at 35 °C, but ultrasound did not enhance the inactivation effect of nisin, in contrast to our findings [11]. However, the cells in their system were likely killed directly by ultrasound (as they used a high-power system), with no injured cells on which nisin could act, thus a lower treatment power might cause damage to the cells making them susceptible to nisin. The present study proves this theory by showing that an injured population exists through flow cytometry (Fig. 2) and that a combined effect of nisin and ultrasound is observed only when nisin is present during sonication i.e. added before ultrasound treatment (Fig. 2, Fig. 3). Although the addition of nisin after sonication is immediate, it is clear that the presence of nisin during sonication is essential for the combination of treatments to have an effect.

### 3.3 System structural effects on E. coli inactivation by ultrasound and nisin

The system viscosity can affect cavitational activity which may also impact inactivation efficacy, as cavitation is believed to be one of the key pathways for microbial inactivation [6]. *E. coli* is studied at 500 kHz only, as inactivation in the liquid system was observed only at this cavitation condition, thus any effect of viscosity (expected to reduce the impact of inactivation) should be visible. Inactivation of *L. innocua* was not continued in the viscoelastic models, as no effect was observed in the liquid system (Fig. 1 a-c).

#### 3.3.1 Rheological characterisation

**Fig. 4:**
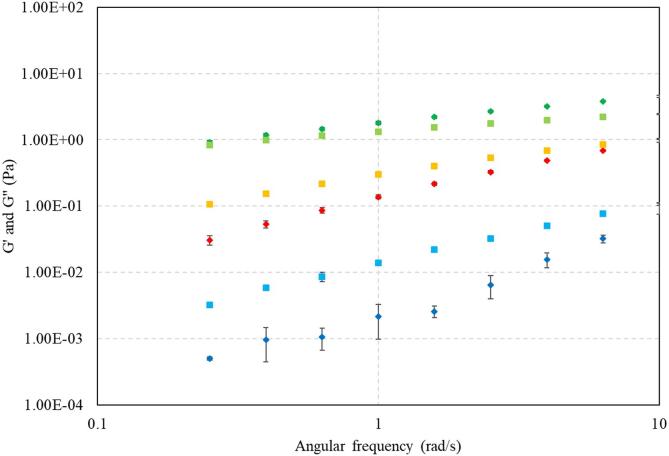
Rheological characterisation of the viscoelastic XG systems. Storage modulus G’ for 0.1% (), 0.3% () and 0.5% () XG, and loss modulus G” for 0.1% (), 0.3% () and 0.5% () XG as a function of the angular frequency. Error bars indicate the standard deviation.

Fig. 4 presents a rheological characterisation of the XG systems at 37 °C, with average values of the rheological parameters G’, G” and tanδ presented on Table 2.

**Table 2:** Rheological parameters for the viscoelastic 0.1%, 0.3% and 0.5% XG systems. Average values for storage modulus G’ and loss modulus G” and their associated standard deviations, and average tanδ values.

**XG concentration (% w/v)**	**Average storage modulus *G’* (Pa)**	**SD**	**Average loss modulus *G”* (Pa)**	**SD**	**tan δ (-)**
0.1	0.017	0.003	0.036	0.001	2.170
0.3	0.331	0.016	0.477	0.004	1.442
0.5	2.424	0.103	1.578	0.037	0.651

For the 0.1% and 0.3% XG systems, the storage modulus *G’* is smaller than the loss modulus *G”*, indicating that the viscous component dominates the flow properties and that the systems behave as liquids (Fig. 4, Table 2). The difference between the moduli reduces as the XG concentration increases, indicating that the elastic component begins to dominate. Indeed, for the 0.5% XG system, the storage modulus *G’* is instead larger than the loss modulus *G”*, indicating that the system becomes more “solid-like” (Fig. 4, Table 2). This increase in the elastic component is also indicated in the tanδ values for the systems investigated: the tanδ for the 0.5% XG system is < 1 thus 0.5% XG can be classified as an elastic solid, and the tanδ of the lower XG concentrations (0.1%, 0.3%) is > 1 thus these are viscous liquids (Table 2). This suggests that there is a transition from liquid to solid between 0.3% and 0.5% XG. Additionally, the XG systems become stiffer/more viscoelastic as the concentration of XG increases (Fig. 4, Table 2), as expected and previously reported [71], [75], [105], [106], i.e. the values of the *G’* and *G”* increase with increased XG concentration. Finally, the frequency dependence of *G’* and *G”* (i.e. gradient) reduces as the XG concentration increases, indicating that the system becomes less fluid and more solid(like) as a result (Fig. 4).

#### 3.3.2 Sonochemiluminescence in viscoelastic systems

**Fig. 5:**
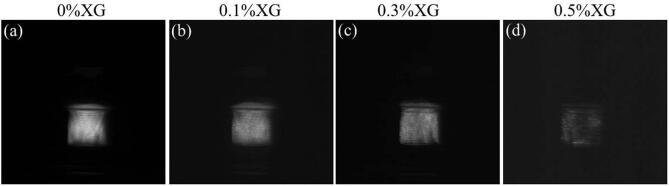
Sonochemiluminescence (SCL) images at 500 kHz of (a) 0%, (b) 0.1%, (c) 0.3% and (d) 0.5% XG systems.

**Fig. 6:**
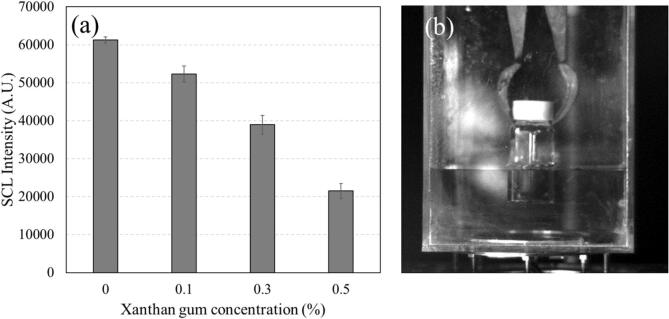
(a) Quantification of SCL intensity, (b) experimental set-up for SCL images. Error bars indicate the standard deviation.

Fig. 5 presents SCL images obtained for 0%, 0.1%, 0.3% and 0.5% XG systems at 500 kHz. Fig. 6 presents a quantification of the SCL intensity and an image of the experimental set-up for SCL imaging. Images and quantification for the other frequencies studied are presented in the Supplementary Information (Figs. S3 and S4).

An effect of system structure is observed: SCL intensity reduces as the XG concentration is increased. A similar reduction in SCL intensity was observed by Gonzalez et al. [37] who obtained SCL images for 0.1%, 0.2%, 0.4% and 0.6% XG systems at 400 kHz with an applied power of 40 W (calorimetric power 28.7 W, similar experimental set-up), although they did not investigate microbial inactivation [37].

#### 3.3.3 The impact of viscosity on the inactivation of E. coli by ultrasound and/or nisin

**Fig. 7:**
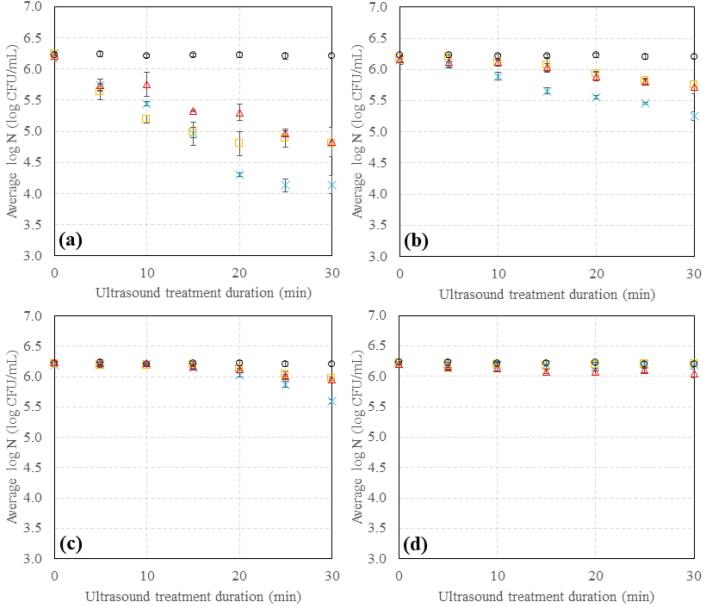
Inactivation kinetics of *E. coli* following 30 min nisin and/or 0–30 min ultrasound treatment at 500 kHz in (a) 0% XG, (b) 0.1% XG, (c) 0.3% XG and (d) 0.5% XG systems. (○) nisin only, () ultrasound only, () nisin then ultrasound, () ultrasound then nisin. Each point is the average of three independent experiments with two replicate samples per experiment. Error bars indicate the standard deviation.

Fig. 7 presents the inactivation kinetics of *E. coli* following ultrasound treatment at 500 kHz, with and without nisin, in the XG viscoelastic food model systems.

A reduced effect of ultrasound is observed on *E. coli* as the concentration of XG (and thus the viscosity of the system) increases, with no effect of ultrasound and/or nisin observed for the 0.5% XG system (Fig. 7). Furthermore, the previously observed effects of different treatment orders on *E. coli* (Fig. 1) are also observed in these higher viscosity systems i.e. enhanced inactivation is observed when nisin is added before ultrasound treatment but not after, for 0–0.3% XG (Fig. 7 a-c). This indicates that the system structure does not impact the efficacy of nisin i.e. that the diffusion of nisin is not hindered through the XG structure at concentrations of<0.3% XG. However, no significant differences in the log reduction are observed between the different treatment methods for *E. coli* in the 0.5% XG system (Fig. 7 d).

As observed in the rheological analysis, a transition from liquid(like) to solid(like) exists between 0.3% and 0.5% XG, explaining the lack of inactivation effect in the 0.5% XG system (Fig. 4, Fig. 7 d, Table 2). Viscosity affects cavitational activity, as bubbles formed during sonication become more stable at higher viscosities and are less likely to collapse [37], [44], [45], reducing the bubble collapse intensity and subsequent ultrasonic effects such as acoustic microstreaming. Thus, we identify the limit at which ultrasound is no longer effective in this system.

There are no existing studies which investigate the efficacy of ultrasound in varied systems of controlled composition and complexity with which the present study can be compared. Furthermore, while studies have been carried out in a range of liquids (growth medium, saline solution, wastewater) and liquid foods (fruit juices, liquid whole egg, milk), the experimental set-up, frequency, microbial species and power intensity varies between them making a useful comparison between systems very difficult [4], [14], [17], [18], [47], [56], [57], [58], [59], [60].

## 4 Conclusions

A systematic study of the effects of combined treatment order (nisin and ultrasound) and system structure in viscoelastic XG-based food model systems, with a comparison between *L. innocua* (Gram positive) and *E. coli* (Gram negative) species was presented. Crucially, the experimental set-up used allowed for a systematic comparison of different cavitation conditions while maintaining all other ultrasonic parameters. *L. innocua* was resistant to ultrasound treatment at the frequencies studied, while ultrasound was only effective for *E. coli* at 500 kHz. The system structure i.e. increased viscosity negatively impacted the inactivation efficacy, most likely by reducing bubble collapse intensity and subsequent ultrasonic effects i.e. streaming.

Enhanced inactivation of *E. coli* was observed for the combination of nisin and ultrasound at 500 kHz, but only when nisin was applied before ultrasound treatment, despite nisin alone having no effect on Gram negative bacteria. Advanced microscopy techniques showed that on a cellular level for the combined treatment, total destruction of the cells occurred while ghost cells were present in systems treated with ultrasound only. Using a combination of kinetics, flow cytometry, microscopy and sonochemiluminescence, the mechanism of ultrasound inactivation was suggested to require a physical aspect resulting in terminal damage to the cell wall and membrane, and the subsequent efflux of cellular contents. Chemical effects are likely to also play a part. Ultrasound in combination with nisin is thought to enhance inactivation by allowing nisin to penetrate the cytoplasmic membrane, but only when nisin is present during sonication. Future work should aim to identify a condition or combination of treatments to which *Listeria* species are not resistant, and incorporate other species such as natural microflora, for a more advanced understanding of ultrasound/nisin efficacy and stress adaptation/resistance. Further optimisation of the ultrasound mode, and investigation of sonochemical versus sonomechanical effects, may also be necessary.

### CRediT authorship contribution statement

**Katherine M. Costello:** Conceptualization, Data curation, Formal analysis, Investigation, Methodology, Project administration, Resources, Writing – original draft, Writing – review & editing. **Eirini Velliou:** Conceptualization, Funding acquisition, Methodology, Project administration, Resources, Supervision, Writing – review & editing. **Jorge Gutierrez-Merino:** Conceptualization, Methodology, Project administration, Resources, Supervision, Writing – review & editing. **Cindy Smet:** Methodology, Supervision, Writing – review & editing. **Hani El Kadri:** Data curation, Investigation, Methodology, Writing – review & editing. **Jan F. Van Impe:** Funding acquisition, Supervision, Writing – review & editing. **Madeleine Bussemaker:** Conceptualization, Methodology, Project administration, Resources, Supervision, Writing – original draft, Writing – review & editing.

## Declaration of Competing Interest

The authors declare that they have no known competing financial interests or personal relationships that could have appeared to influence the work reported in this paper.
