# Supplementary material for: The effect of ultrasound treatment in combination with nisin on the inactivation of Listeria innocua and Escherichia coli
Source: Ultrason Sonochem. 2021 Oct 7;79:105776. doi: 10.1016/j.ultsonch.2021.105776 (PMC8560821; doi:10.1016/j.ultsonch.2021.105776)
Supplement: Supplementary data 1 [file mmc1.docx]

**The effect of ultrasound treatment in combination with nisin on the inactivation
of *Listeria innocua* and *Escherichia coli***

Katherine M. Costello, Eirini Velliou, Jorge Gutierrez-Merino,

Cindy Smet, Hani El Kadri, Jan F. Van Impe and Madeleine Bussemaker

# Supplementary Material

A schematic showing the experimental set-up is presented in Figure S1.


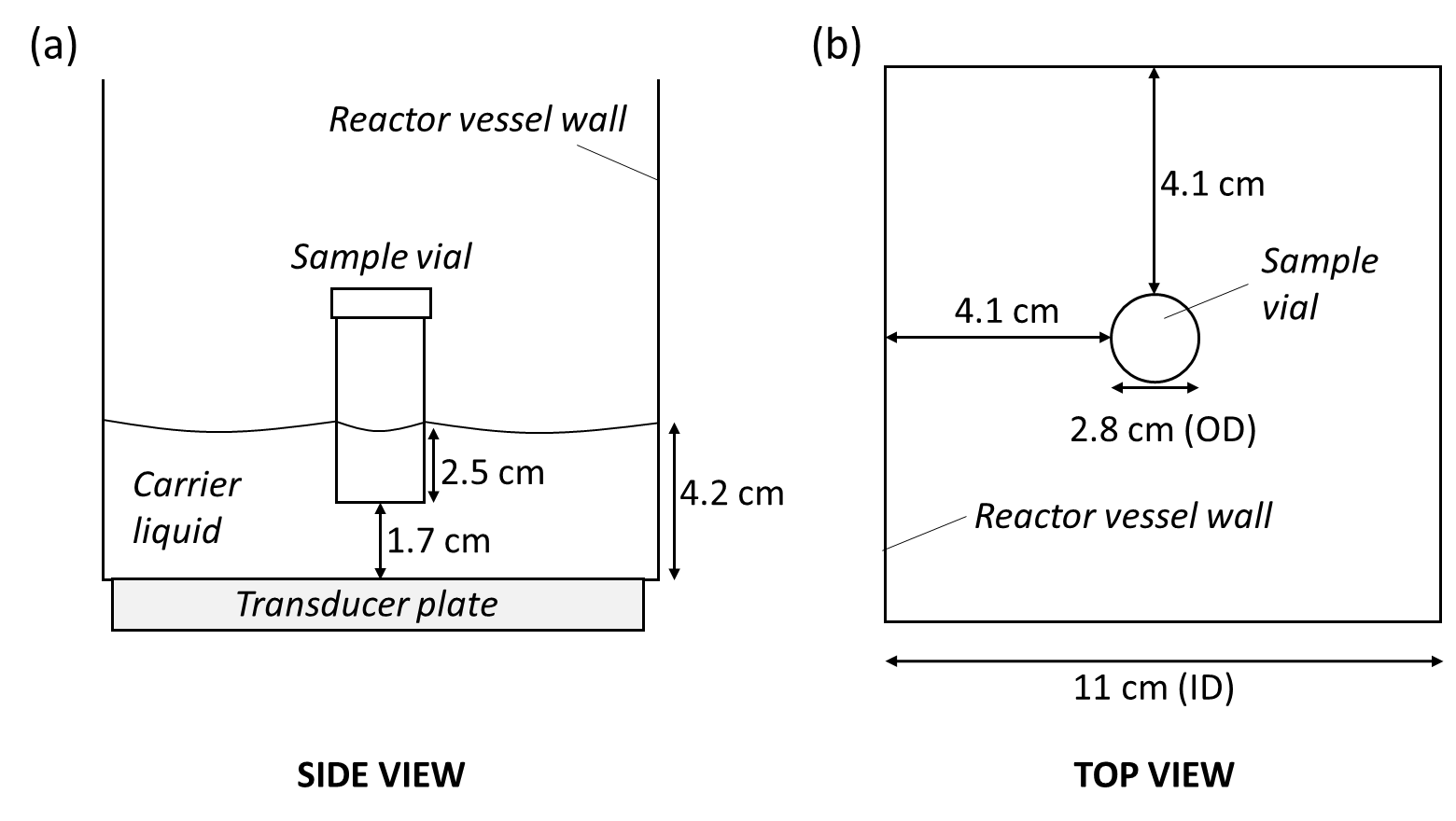


**Figure S1**: schematic showing the experimental set-up and dimensions inside the reactor vessel with (a) side view and (b) top view. The menisci shown in (a) are exaggerated for clarity. ID: internal diameter. OD: outer diameter.

Figure S2 displays the ultrasonic system characterisation (calorimetry, dosimetry).


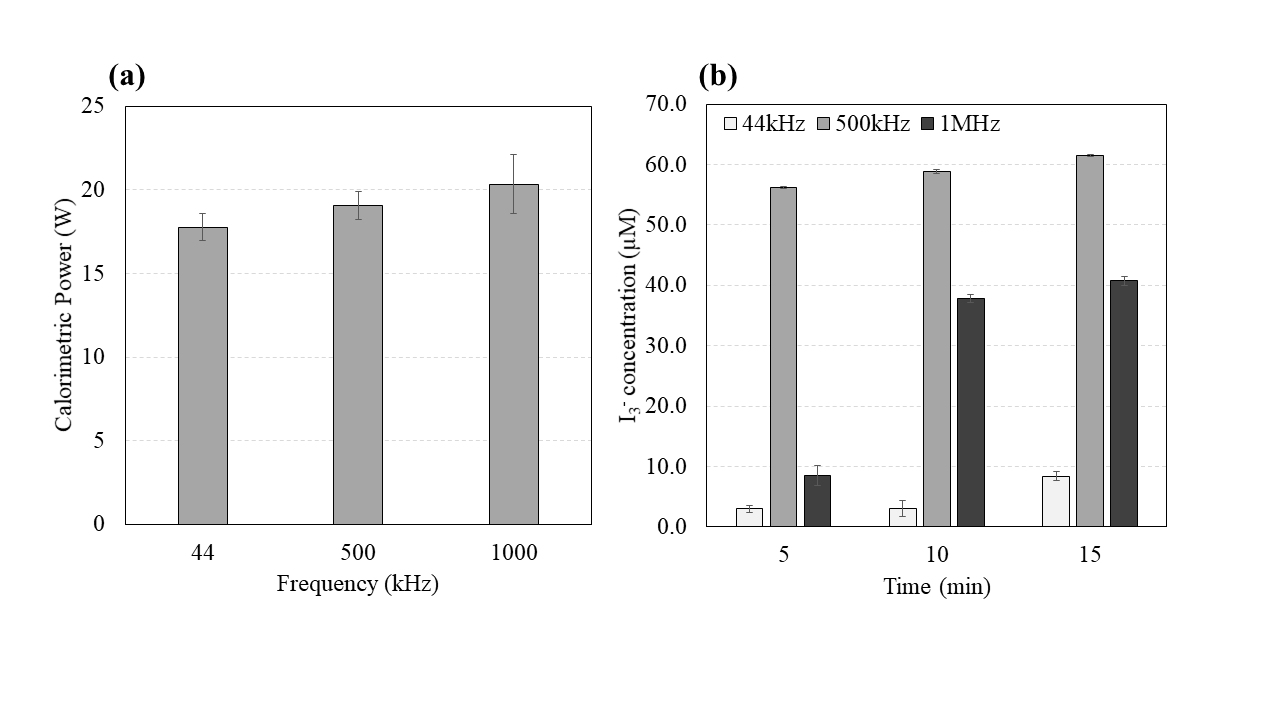


**Figure S2**: (a) calorimetric power applied to the system and (b) concentration of I_3_^-^ as a result of KI dosimetry. In both studies, an applied power of 30 W was used.

Figure S3 displays sonochemiluminescence (SCL) images at 44 kHz, 500 kHz 1000 kHz, to supplement Figure 5 of the manuscript. Figure S4 is a quantification of the SCL intensity at the frequencies studied and supplements Figure 6 of the manuscript. For both figures, the results at 500 kHz are reproduced from the manuscript for ease of comparison with other frequencies.


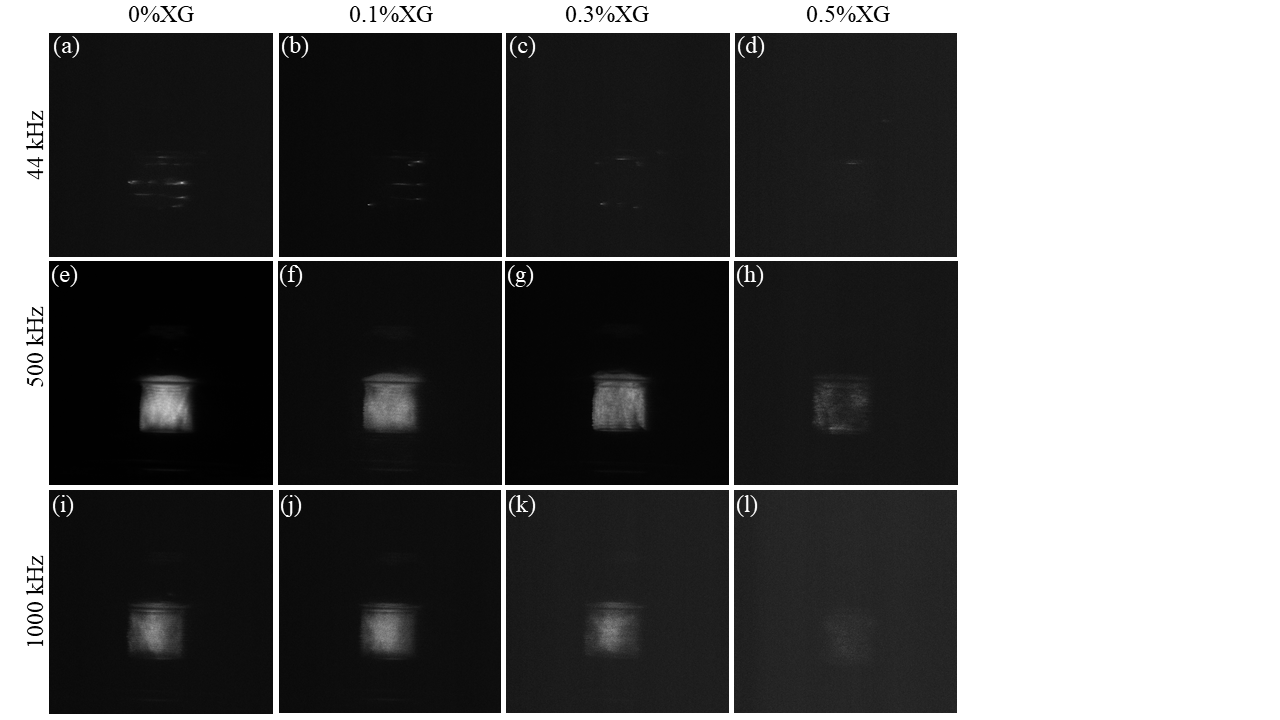


**Figure S3:** Sonochemiluminescence (SCL) images at 44 kHz, 500 kHz and 1000 kHz of (a,e,i) 0%, (b,f,j) 0.1%, (c,g,k) 0.3% and (d,h,l) 0.5% XG systems.


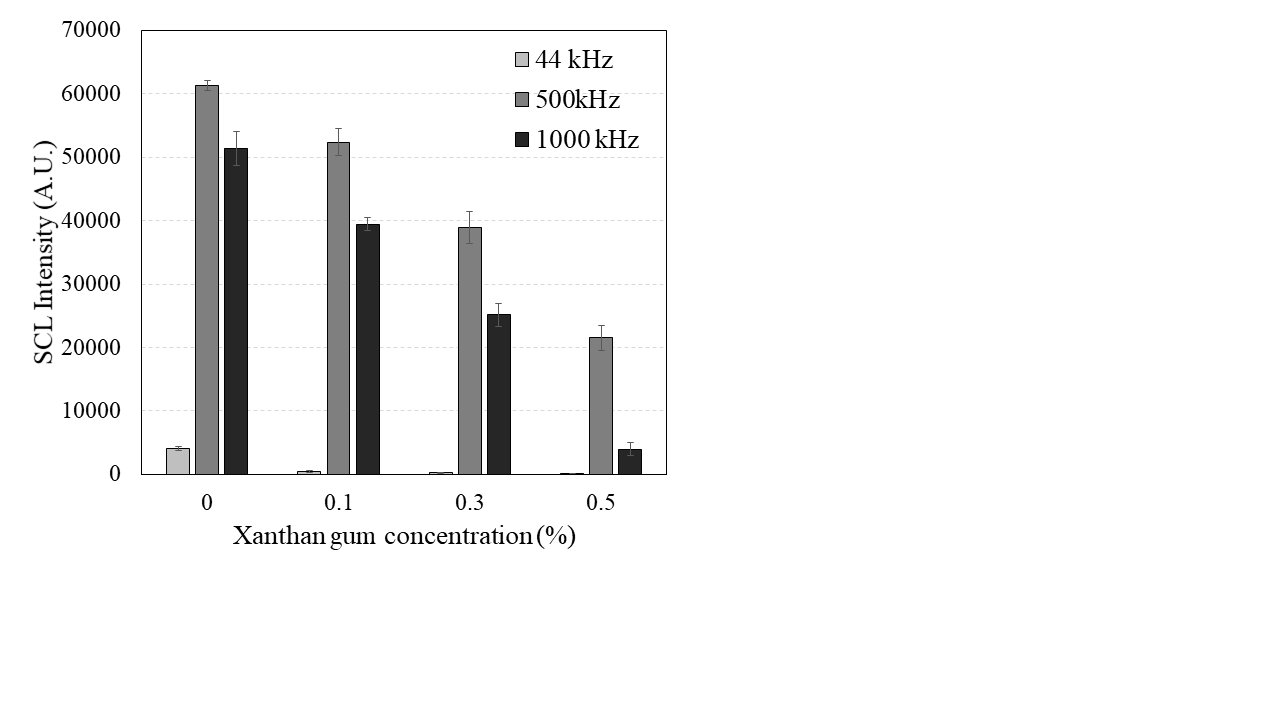


**Figure S4:** quantification of SCL intensity at 44 kHz, 500 kHz and 1000 kHz with varied XG concentration (0 – 0.5%).
